# A novel tissue-specific meta-analysis approach for gene expression predictions, initiated with a mammalian gene expression testis database

**DOI:** 10.1186/1471-2164-11-467

**Published:** 2010-08-11

**Authors:** Kshitish K Acharya, Darshan S Chandrashekar, Neelima Chitturi, Hardik Shah, Varun Malhotra, Sreelakshmi KS, Deepti H, Akhilesh Bajpai, Sravanthi Davuluri, Pranami Bora, Leena Rao

**Affiliations:** 1 Institute of Bioinformatics and Applied Biotechnology (IBAB), Biotech Park, Electronic City, Bangalore - 560100, Karnataka state, India; 2 Shodhaka Life Sciences Pvt. Ltd., IBAB, Biotech Park, Bangalore - 560100, India

## Abstract

**Background:**

In the recent years, there has been a rise in gene expression profiling reports. Unfortunately, it has not been possible to make maximum use of available gene expression data. Many databases and programs can be used to derive the possible expression patterns of mammalian genes, based on existing data. However, these available resources have limitations. For example, it is not possible to obtain a list of genes that are expressed in certain conditions. To overcome such limitations, we have taken up a new strategy to predict gene expression patterns using available information, for one tissue at a time.

**Results:**

The first step of this approach involved manual collection of maximum data derived from large-scale (genome-wide) gene expression studies, pertaining to mammalian testis. These data have been compiled into a Mammalian Gene Expression Testis-database (MGEx-Tdb). This process resulted in a richer collection of gene expression data compared to other databases/resources, for multiple testicular conditions. The gene-lists collected this way in turn were exploited to derive a 'consensus' expression status for each gene, across studies. The expression information obtained from the newly developed database mostly agreed with results from multiple small-scale studies on selected genes. A comparative analysis showed that MGEx-Tdb can retrieve the gene expression information more efficiently than other commonly used databases. It has the ability to provide a clear expression status (transcribed or dormant) for most genes, in the testis tissue, under several specific physiological/experimental conditions and/or cell-types.

**Conclusions:**

Manual compilation of gene expression data, which can be a painstaking process, followed by a consensus expression status determination for specific locations and conditions, can be a reliable way of making use of the existing data to predict gene expression patterns. MGEx-Tdb provides expression information for 14 different combinations of specific locations and conditions in humans (25,158 genes), 79 in mice (22,919 genes) and 23 in rats (14,108 genes). It is also the first system that can predict expression of genes with a 'reliability-score', which is calculated based on the extent of agreements and contradictions across gene-sets/studies. This new platform is publicly available at the following web address: http://resource.ibab.ac.in/MGEx-Tdb/

## Background

Establishing gene expression patterns would facilitate understanding of almost every aspect of cellular and molecular biology. Since mass scale detection is easier in case of mRNAs than proteins, many studies have considered gene expression determination at the transcript level. These reports have resulted in large number of signature sets of genes corresponding to various experimental/physiological conditions and tissues/cell-types of multiple species. The phrase 'gene-sets' is used in this article to refer to the data-sets, which usually have a large list of genes with additional expression values, as well as the simpler gene-lists, which are a smaller set of genes, and usually without additional data.

Many databases have been created using a variety of gene-expression data. The main types of data considered by such databases include: Expressed Sequence Tags (ESTs) (e.g., *UniGene *[1]), microarray (e.g., *BioGPS, earlier SymAtlas *[2]), Serial Analysis of Gene Expression (SAGE) (e.g., *GermSAGE *[3]) and manually curated information from different small scale experiments (e.g., Human Protein Reference Database or *HPRD *[4]). The majority of these databases can extract and display the expression-related data for genes in multiple species, tissues and conditions. However, our preliminary observations showed that the transcription patterns of genes revealed by different databases frequently contradict each other.

Further, the reproducibility of data from microarray is questionable in spite of the fact that this is the most common expression profiling technique used [5-9]. Hence, it is preferable to derive a 'consensus' expression status for genes by comparing the data-sets from comparable conditions and tissues/cell-types, rather than relying on one study only. It is possible to derive a simple expression status (expressed or not expressed) for conditions or tissues, for which multiple data-sets are available. However, such simple expression profiles cannot be retrieved by the existing databases, even when the expression data for the corresponding conditions/locations is available. This is because of the following reasons:

a) Some of the published microarray data do not seem to be covered by the existing repositories/databases. For example, the raw data is not deposited in a significant number of cases [10]. The smaller gene-lists, which are sometimes the only gene-sets reported for specific conditions/cell-types, are particularly ignored by most databases, with exceptions such as Oncomine [11], which focuses on human cancer data alone. The microarray data corresponding to some studies such as the effects of androgen deprivation [12], FSH treatment [13], and testicular carcinoma [14] were not found in most of the resources.

b) While most existing databases permit querying with gene symbols (e.g., PRM1) or complete names (e.g., protamine 1), very few databases allow the user to successfully use the names of cell types, tissues, or specific physiological conditions, as query terms. More importantly, whenever such queries are allowed, the corresponding results do not seem to be reliable (Acharya *et al*, unpublished).

c) Many databases focus on the relative expressions of genes across two conditions and/or the level of expression of genes. This type of information is difficult to compare across data-sets. Hence, most existing databases simply provide access to the original data, or represent it graphically, rather than making a final statement about the expression/transcription status of genes under various conditions. Somehow, the value of a simple expression statement in a binary form (i.e., expressed or not expressed) is apparently ignored by most existing databases.

In view of such limitations in the available online resources, we have initiated a novel approach to create tissue-specific databases by making maximum use of the available information and facilitate the prediction of gene expression patterns under various conditions.

The current article reports creation of the first of its kind tissue-specific database for predicting gene expression patterns. Our objective was to collect maximum gene expression data-sets corresponding to a specific tissue, and enable identification of genes with consistent expression status across multiple studies corresponding to specific conditions/cell-types. The consensus expression status derived for genes using such an approach was expected to largely agree with manually curated data (MCD), particularly in cases where the information is supported by multiple small scale studies. The performance of the new database was compared with several existing resources. Testis, where the cell differentiation is unique among adult tissues, was taken as the first tissue for database creation.

## Methods

The strategy can be summarized as follows: a) Collect maximum amount of genome-wide expression data, including smaller gene-lists reported in the manuscripts, corresponding to the mammalian testis tissue. b) Create a database using these data. The database would have multiple gene-sets corresponding to the same or similar physiological and/or experimental conditions. c) Measure the extent of agreement or contradictions for each gene's expression status, across comparable gene-sets in the database. d) Use this degree of consistency to identify the 'consensus' expression status for each gene, for maximum possible conditions/locations, and derive a 'reliability-score' for each consensus expression status. e) Create interfaces that would allow users (of the database) to easily obtain lists of genes for various testicular conditions/cell-types, and also differentiate the genes with higher reliability, from those with low reliability scores, for a specific expression pattern.

### Collection and curation of data

Lists of genes reported to be transcribed or dormant following genome-wide expression studies in testis or specific testicular cells (spermatogonia, spermatocytes, spermatids, spermatozoa, Sertoli cells, Leydig cells and myoid cells) under different conditions were collected. Two approaches were used to collect such gene-sets, from studies on mouse, rat and human species (details of the process in additional file 1):

a) Literature search was performed to collect relevant articles. A search strategy was carefully designed to select appropriate query terms, search fields and combinations. The initial PubMed results were filtered by screening the title of the articles. Abstracts of the short-listed citations were read to identify articles of probable relevance (articles that might report expression of multiple genes in mammalian testis or its cell types). These articles were then searched for the list of genes reported to be expressed, up-regulated, down-regulated, etc. Original complete lists reported in the supplementary notes (such as the journal web-sites or the author's own page) were traced. When this was not possible, the genes reported to be expressed were extracted from the main text of the published article.

b) Microarray repositories such as Gene Expression Omnibus (GEO) [15], ArrayExpress [16], Oncomine, Stanford Microarray Database (SMD) [17] and Center for Information Biology gene EXpression database (CIBEX) [18] were screened for data-sets related to mammalian testis. The search and collection of data-sets were performed manually. The data-sets were uploaded into the new database along with associated details such as the statistical methods and platforms used in the experiments, using a specially designed excel-based format. Newly created programs (please see below) extracted the contents of such files, including genes and their expression status, into designated tables of the database. Author/depositor-calls on the expression status were used. No additional analyses were performed.

Each gene-set was collected along with the necessary cognate information, such as,

1. the expression status (expressed or dormant),

2. species,

3. tissue-area or cell type, and

4. specific physiological or experimental condition.

This set of basic parameters is henceforth referred to as 'Expression Status under specific Location and Condition (ESLC)'. The 'conditions' include normal physiological state, diseases, developmental stages and treatment with hormones and/or other chemicals. This type of information and the corresponding gene-set were systematically formatted and entered into the database. No attempts were made to perform fresh statistical analysis. Instead, the 'expressed' or 'not expressed' call by the author(s) was relied upon. In any study with multiple hybridizations, the expression status indicated in the majority of cases was used as the final expression status. For example, any gene reported to be 'expressed' in the majority of the hybridizations/samples was identified as 'expressed'. In several cases, the gene-sets reported by the authors had to be split into multiple gene-lists (see additional file 1 for an example). Thus, several gene-sets were collected, each ranging in size from three genes to several thousand genes. Each gene-set was characterized by the transcription, or lack of it, in specific location and condition.

### A scoring system was used to suggest the degree of reliability of the ESLC

For every ESLC, a score was derived for each gene and added up across comparable gene-sets to indicate the reliability of that ESLC. This 'reliability score' reflected the consensus expression status across multiple gene-sets. Each gene was assigned a score of two for a specific and definite expression status (transcribed or dormant). When the evidence for an ESLC of a gene increased, across the gene-sets, the score increased. For example, a gene reported to be 'not detected' in normal rat testis by two studies, would be shown as 'dormant' in normal rat testis with a reliability-score of four. Thus, a lower score would indicate either lack of supporting evidences or presence of contradicting reports for the specific expression status under consideration (details in additional file 2).

### Database creation (see additional file 3)

MySQL Relational Data-Base Management System (RDBMS) was employed for storing data. A table was dedicated to store the basic gene-related information including the gene name, locus and transcript details. Another table was used to store gene identifiers such as gene name, gene description, official gene symbol and the National Center for Biotechnology Information (NCBI) gene identifier etc. There are also tables corresponding to each main expression condition: each table was created to store the genes with the derived expression status along with details pertaining to their ESLC's.

Perl based CGI script has been used to create an interface for entry of gene-lists. Programs were written for automatic downloading of basic information for every new gene entered into the database. Another program used the available identifier for each gene in the uploaded gene-set, and retrieved its respective NCBI gene identifier and the gene symbol. After the basic information compilation, the genes were put in queue for further information downloading from online resources, followed by uploading into the new database. Specially designed Perl programs such as LWP modules (http://search.cpan.org/~gaas/libwww-perl-5.836/lib/LWP.pm) were used to connect to NCBI and, with the aid of NCBI E-utilities (http://eutils.ncbi.nlm.nih.gov/entrez/query/static/eutils_help.html), the required information was downloaded. The information included the NCBI gene id, official gene symbol, aliases, gene sequence, gene summary, chromosomal location, potential promoter sequence [-1000 to +200 bp] and all transcript sequences (along with exon-intron details) corresponding to each gene. Using gene symbol and Swiss-Prot IDs downloaded from NCBI gene database, protein-related information encoded by that gene was downloaded from Swiss-Prot [19] (http://ca.expasy.org/sprot). Similarly, transcription start sites were downloaded from dbTSS [20] (ftp://ftp.hgc.jp/pub/hgc/db/dbtss/). When the information was not available in dbTSS for a gene, the 5' end of corresponding NCBI RefSeq sequences (transcripts) was used to represent the TSS position. Perl codes were written to ensure automatic incorporation of the downloaded data into the database.

### Comparison of MGEx-Tdb with other existing resources

Initially several databases were considered for comparison (details in additional file 4). But only UniGene, BioGPS and HPRD were selected and compared in detail as described below.

### Assessing the degree of agreement of gene expression status between the databases and the manually curated data (MCD)

Literature search was performed to compile reports on expression status of 60 human genes, which were randomly chosen (to avoid bias, MGEx-Tdb was not used as source). Selected articles corresponding to these genes were read in detail and evidences were collected for certain ESLCs. The focus was on studies that addressed the expression of one or a few genes only. Mass scale studies such as genome-wide expression profiling studies, which were used to create MGEx-Tdb, were avoided. More than one independent experimental evidence for the same expression status, by at least two separate studies, were available for 13 of the 60 chosen genes (see additional file 5).

The expression status was obtained from the newly developed database as well as from UniGene, BioGPS and HPRD, for each of the 13 genes. This information was compared with the unanimous expression pattern derived from MCD, and scores were then assigned to indicate the extent of agreement (see additional file 6 for scoring details). Each database was thus scored for concurrence with MCD for the 13 genes. This exercise was performed independently by two individuals and the results were concurrent (see additional file 7 for details).

### Coverage of the databases

The number of genes for which the databases could provide expression status was noted. A total of 110 human genes were used (i.e., 50 more randomly chosen genes added to the list of earlier selected 60 genes). Every database was given a score of one when a gene was present in the database, and an additional score of one if the expression information for that gene is also present in the database. Thus, the maximum total score a database could get was 220.

### Final *in silico *validation of the database (see additional file 8 for details)

Ten different genes reported to be predominantly expressed in mammalian testicular cells were taken and compared for two aspects as described below.

a)	Relative amount of information per gene: Every database was scored for availability of information for each gene's expression in testis, in specific cell type, condition and in different species (mouse, rat and human).

b)	Agreement of each gene's information from database with that of the information available in the literature: For this comparison, a procedure similar to the one used above (in the case of MCD vs. database comparison) was employed.

## Results

A database with a total of 62,185 genes has been created. The genes were derived from 769 gene-sets, which in turn were collected from online resources like ArrayExpress, GEO as well as, from publications (table 1). The new database provides 19 different ESLC for humans (25,158 genes), 74 for mice (22,919 genes) and 26 for rats (14,108 genes).

**Table 1:** The contribution of the number of gene-sets from each resource across species.

	No. of gene-sets*		
	
Resources	Human	Mouse	Rat
ArrayExpress	36 (15537)	9 (11438)	45 (10452)
GEO	131 (6337)	302 (6359)	24 (8594)
PubMed	43 (225)	138 (3791)	41 (1116)

Total	210 (7366)	449 (7196)	110 (6721)

*The value in parenthesis represents average gene count across the gene-sets.

While cancer-related mass scale gene expression profiling have almost been exclusive to humans, studies on hormone treatment and gene knock-outs were more common in mice. Chemical treatment studies have been very frequent in rats. In addition, the newly created platform has the potential to indicate the expression pattern of a gene in about 36 different tissues under normal conditions.

**Figure 1:**
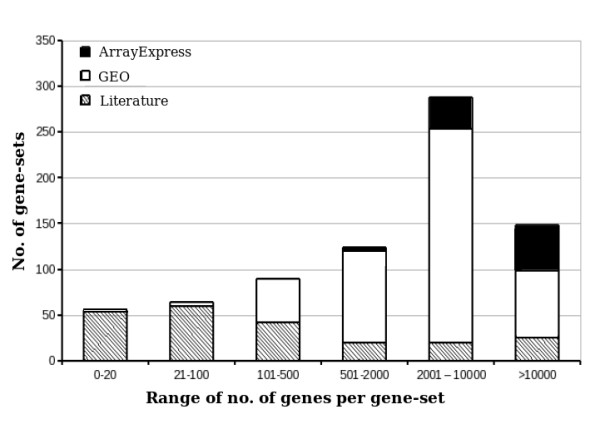
**Categorization of gene-sets in MGEx-Tdb based on the 'number of genes per gene-set'. For example, there are 52 small gene-sets (each with less than 20 genes) and of these, 51 sets have been retrieved from literature**.

The gene-sets in the new database are predominantly transcript-lists from microarray studies. Mass scale studies were rare for testicular cell types in humans unlike the rodent species. The majority of the gene-sets uploaded in the database have more than 500 genes each, but smaller gene-lists reported in manuscripts, have also been included in this database (Figure 1).

Multiple gene-sets were indeed collected for certain conditions. For example, 124 gene-sets were collected for testicular cancer in humans from 7 reports, 35 gene-sets for normal human testis from 13 reports and 6 gene-sets for mouse spermatids from 5 reports. There are about 21 ESLCs, for each of which at least 2 different studies were compiled. Such comparable gene-sets were particularly useful in deriving a consensus of expression patterns and reliability-scores. However, there were many cases where we could get only one research article per condition such as adjudin and cadmium chloride studies in rat, with 6 gene-sets each.

During the comparative study, HPRD showed a better agreement with MCD (as shown in table 2) than other databases. However, it had the lowest coverage for human genes among the databases considered, with a score of 204. UniGene obtained 219, whereas MGEx-Tdb and BioGPS got the maximum possible 220 for coverage. It should be noted that HPRD is mainly a human protein database, with focus on multiple aspects of proteins. Unlike other databases considered, HPRD provides the expression status that is established by manual curation of small-scale studies, at protein as well as mRNA levels. Hence, the higher agreement (see tables 2 and 3) with MCD as well as the lower coverage of HPRD is not surprising.

**Table 2:** Extent of agreement for gene expression pattern of 13 genes, between MCD and the databases.

Database	Score (maximum = 26)	% agreement with reports on individual gene studies
HPRD	20	77
MGEx-Tdb	14	54
UniGene	12	46
BioGPS	11	42

**Table 3:** Relative assessment of amount and details of information, and agreement with MCD.

Database	Information availability and volume of supporting data		Agreement with reports on individual gene studies	
	
	Score (maximum = 33)	% score	Score (maximum = 11)	% agreement
HPRD*	13	39	8	73
MGEX-Tdb	27	82	5	45
UniGene	14	42	3	27
BioGPS	11	33	5	45

Notes:

See additional file 8 for detailed methods;

*HPRD is specific for humans. Hence the score depicted for this database is not an average across the species.

MGEx-Tdb performed better, than other databases considered, in predicting the expression status of genes, when queried with various physiological conditions (see table 4, also see additional file 9 for methods). Even when queried with specific genes, MGEx-Tdb provided much more details (see table 3 and additional file 8). This new database also provided transcription information for more genes in the context of developmental stages, compared to the databases specialized for development-related gene expression patterns (see table 5, also see additional file 9 for methods).

**Table 4:** Results in response to queries for expression status of genes under different testicular conditions.

	No. of genes retrieved in response to different queries					
	
Database^1^	Normal testis (human)	Azoospermia (human)	Asthenozoospermia (human)	Testicular cancer^2 ^(human)	Adjudin treatment (rat)	Developmental stage-postnatal (mouse)
MGEx-Tdb	12753	5215	10	16617	10982	15209 *(day 14)*
BioGPS^3^	403	14	0	3	0	2
RefExA^4^	92	3	0	3	NA	NA
TissueDistributionDBs^4^	16124	4	0	2	0	0
UniGene^4^	18421	4	0	194	0	1^5^
HPRD	4249	2	0	0	NA	NA

Notes:

See additional file 9 for detailed methods;

NA: Not Applicable, i.e., data restricted to humans only in these databases;

^1^The URLs are listed in the additional file 4.

^2^Querying with testis AND cancer retrieved 116 genes in BioGPS; "Germ cell cancer" in testis retrieved 9655 genes (10042 hits) in TissueDistributionDBs and "Germ cell tumor" in testis retrieved 14874 genes (18130 hits) in UniGene and 2 genes in HPRD.

^3^Includes results from human, mouse & rat species (search can not be restricted to specific species).

^4^Number of genes in the results corresponding to different queries: probe names in case of RefExA & cluster IDs in case of UniGene & TissueDistributionDBs.

^5^Querying with alternative equivalent terms of postnatal (neonate) retrieved 10467 genes (11519 hits) in UniGene.

**Table 5:** Results for expression status of genes at developmental stages in mouse testis from different databases.

Databases^1^	Postnatal period	Specific postnatal Stage (0-6 day/TS27)
MGEx-Tdb	21016^2^	21195
Bgee	15899	15501
MGI (GXD)	5954^3^	NA
MRG	~8500	~8500
4DXpress	ND	ND

Notes:

See additional file 9 for detailed methods;

NA: Not Applicable, i.e., no query feature available for early post-natal stages of development; ND: No Data in postnatal development stage [available till TS 26 only]; TS: Theiler stage (A term used to denote the stage of development of a mouse; Theiler, 1989); **Bgee**: a data**B**ase for **G**ene **E**xpression **E**volution; **MGI (GXD)**: **M**ouse **G**enome **I**nformatics (**G**ene E**x**pression **D**atabase); **MRG**: **M**ammalian **R**eproductive **G**enetics; **4DXpress**: E**Xpress**ion database in **4D **(four dimensions).

^1^The references and URLs are listed in the additional file 4.

^2^MGEx-Tdb results correspond to genes transcribed/dormant on specific days between day 0-20 and many genes cancel due to contradictory results. However, one can obtain better results for specific stage using this database, as indicated in the third column.

^3^The results include some repeats.

As expected, the tissue-specific compilation of mass-scale gene expression data resulted in agreement with MCD (individual gene studies, see table 2). MGEx-Tdb was as good as, or better than, UniGene and BioGPS, in terms of agreement with MCD. Since the results from HPRD agreed completely with MCD for 8 of the 13 query genes, it attained the highest % score for agreement with MCD as shown in table 2. This is despite the fact that HPRD showed only partial agreement with MCD for 3 genes, did not provide expression-related information for 1 gene and lacked any information for another.

The newly created platform is also able to successfully separate the genes, which show consistent transcription status across the gene-sets/studies from those which do not. For example, using the output of MGEx-Tdb in case of testicular cancer, one can not only identify 8,410 transcribed and 8,207 dormant genes, but also categorize them further based on the reliability-score. Of the 8,410 genes, 674 had a reliability-score greater than 70, indicating that they have a higher probability of being expressed in human testicular cancer conditions. Similarly, 2,729 genes had a reliability-score between 10 and 70, while 1555 showed a score of 4 or less. In another example of 7,570 genes transcribed in 14 day old mice, 875 had a reliability-score of ≥6 and 2291 genes had a score of 2.

Overall, MGEx-Tdb could offer the following advantages over the other databases analyzed: a) more useful query features, b) reliability of information provided, as indicated by high agreement with MCD, c) better output in terms of the number of genes, and d) the unique reliability indicator for each gene, under different ESLC. This new database did not agree with MCD to the same extent as HPRD, but showed better coverage and higher number of genes in the output for disease-related query.

An overview of MGEx-Tdb usage is provided in the form of screen-shots in the additional file 10.

## Discussion

Researchers have been trying to compile and compare microarray data from different studies. ArrayExpress, TranscriptomeBrowser [21], Genevestigator [22] and COXPRESdb [23] are some examples. Variations in experimental conditions across studies have hindered such efforts. Recommendations for systematic reporting of experimental conditions [24,25] and new methods for cross-platform comparisons of microarray results [9,26] have improved our ability to make use of the available microarray data. However, many microarray data-sets are not comparable due to various reasons. These reasons include non-compliance with MIAME [27] and complications in statistical methods of data processing, particularly when the studies have used different microarray platforms [7]. There are also a large number of reports where raw data is not available and only selected genes with specific expression pattern are listed. Such gene-lists lack the necessary basic information for comparing expression level-related information.

The method reported here allows comparison of the gene-sets irrespective of associated information such as intensity values, statistics, platform and probes. Obviously, this simplification would mean loss of other important information such as relative up/down regulation and levels of expression. Many 'meta-analysis' approaches have considered such details of expression data [28,29], but they are applicable to raw data only. It is also difficult to find consensus by the traditional methods. For example, if a study (A) finds gene xyz to be expressed very high in condition 1 (C1) compared to condition 2 (C2), study B finds xyz to be expressed only marginally higher in C1 than in C2, and study C finds it to be expressed almost equal in both C1 and C2 - they all disagree with each other as far as the 'relative levels of expression' is considered. The novelty of our approach is that, by considering only the 'transcribed' or 'dormant' status, the data could be compiled across different microarray platforms, and we can state that gene xyz is 'present' in C1. Thus, this method of comparing expression status in a binary form allows use of most of the available microarray data, including simple gene-lists.

In fact, obtaining a list of genes expressed or not expressed in specific conditions, and deriving consensus across studies, can provide an extremely important alternative to biomarker identification. Generally, the genes that are up regulated or down regulated and those whose function/ontology is well established are considered as possible markers. With the new approach one can compare the list of genes with high reliability score for the 'expressed status' under a normal condition with a similar set of genes absent in an abnormal condition. The union list derived after such a comparison would have a unique value as a set of potential biomarkers. Similarly, genes that are more likely to be dormant in normal conditions, but expressed in abnormal tissues would also be important. We are currently trying to use this approach to identify genes that have a strong correlation with azoospermia.

Nevertheless, the current database can also be used as a single source for identifying most of the mass scale gene expression data as it directs the user to the original data in all cases. Those interested in using the original microarray data can do so, and perform their own comparison and analysis.

Approaches similar to the one used in this study have been used for other purposes earlier too: Smith *et al*. [30] applied such a method for meta-analysis of breast cancer microarray data and Harsha *et al*. [31] for identifying potential pancreatic-cancer biomarkers. Very recently, Culhane *et al. *[32] also reported a very similar approach to create a gene expression database, GeneSigDB, which considers gene lists from tables or figures embedded in publications or included as supplementary material on the journal's or the author's website. But, GeneSigDB does not use raw data, cover testis-related conditions or derive a consensus across data-sets (from different studies). New methods such as Gene Set Enrichment Analysis (GSEA) [33], Parametric Analysis of Gene set Enrichment (PAGE) [34] and Generally Applicable Gene set Enrichment for pathway analysis (GAGE) [35] process data across multiple data-sets in such a way that, the specific details of data-processing within each study are not required to bring out meaningful information from the microarray experiments. However, the objective of GSEA and PAGE was to gain insights into biological mechanisms by clustering genes across studies, while our focus was in deriving the consensus information along with a reliability score.

Compilation of gene-sets corresponding to comparable conditions and locations, and deriving a reliable ESLC for each gene, can be useful in various ways. One can cluster genes based on their expression pattern in different ESLCs. Such clustering can help to identify genes having strong association with specific conditions and/or locations. For example, genes with consistent expression in normal testis but absent in infertility conditions might be of significance for researchers. The higher the reliability-score of a gene, the higher will be its chances of being a biomarker and/or a candidate for research in diagnostics, prognostics and therapeutics. Moreover, tissue-specific databases, such as MGEx-Tdb, also have the potential to assist in exploring the variation or conservation of expression of genes across different species in multiple tissues.

The need for systematically compiling gene-expression data in one place is obvious from previous efforts. In fact, TisGeD [36], a new database, has been reported during the last stages of the writing of this manuscript. This database is a compilation of data for most tissues and species, mainly from existing databases. But it seems to have failed to make the best use of all available information, at least for the testis tissue. On the contrary, an effort like the current one may not be always practical. The biocuration process consumed a significant amount of time (about 3 years) and is eventually limited to only one tissue. However, it would provide more reliable information. There is perhaps a compromised approach possible. While about 222 gene-sets in the database were retrieved from literature, 156 of them had less than 500 genes per set. By avoiding such smaller gene-sets, one might save time - albeit with some loss of information.

Even though this study has compared MGEx-Tdb with a few well-established databases, the purpose is of course not to undermine the value of these pre-existing resources. Such databases have their own specific advantages and, in many cases, a wider variety of applications. The objective of comparing the different databases was to validate the novel approach.

While MGEx-Tdb can facilitate unique applications in the gene expression studies in the context of mammalian testis, it has a few limitations and there is a scope for further improvement in different aspects. For example, incorporation of level of expression along with the basic expression status might be possible in many cases. The method of calculating 'reliability-scores' for the expression patterns can be improvised by considering the details such as sample size and validation of the microarray data, reported along with gene-sets. Factors such as unavailability of complete data in many cases, diversity in analytical methods used, and lack of experimental details in many of the published gene expression studies have been major hurdles for the compilation of parameters mentioned above. Nevertheless, we are already making attempts to make the possible improvements. We are also trying to include data from other types of mass scale studies. In the current database, we have used the non-microarray data in some cases only, particularly when a list of genes was reported in the manuscript or in the supplementary notes. The data in the repositories could not be included due to complications in the process of converting the unique identifiers (e.g., SAGE tags) to standard gene names or ids. We shall complete these tasks in a revised version of the database. Moreover, efforts are on to include data from more mammalian species for the testis tissue, further improve query features of this database and even develop a few other tissue-specific databases.

Most of the existing data permit only predictions, rather than actually establishing a final expression status for different genes. This can be explained as follows: a) There is a larger amount of data available for the expression of genes at the RNA level, compared to protein level, and transcription doesn't guarantee continued translation into proteins. Thus, the mRNA data can only be used to suggest or predict the expression of genes into final proteins. b) The expression status of some genes can vary across samples, even within a study. The genes which behave the same way across samples and studies are more likely to have a stronger association with the physiological condition of the tissue/cell type of interest. This means, the data can only be used to predict the expression possibilities. And, it will be useful to 'predict' expression patterns of genes, using a reliability-score such as the one reported here.

## Conclusions

We demonstrate that manual collection of mass scale gene expression data will allow derivation of a 'reliability score' for binary expression status of genes. The simplicity of the new approach permits consideration of expression data obtained using ESTs, SAGE, Massively Parallel Serial Sequencing (MPSS), proteomics and other techniques as well. This way, maximum amount of existing data can be used for better prediction of gene expression patterns across a variety of reports. We also report the mammalian testis specific gene expression database, which performed better than most other gene expression databases in various aspects. MGEx-Tdb is the first database that attempts to make maximum use of the available data to provide a quantitative indicator for the expression-probability of genes under multiple conditions and locations, in the context of at least one mammalian tissue. We are further improving the scoring method and enhancing the volume of relevant gene expression data. MGEx-Tdb can be used for easy retrieval of information about expression of genes in several conditions of testis or its cell types in 3 most well-studied mammalian species. This in turn can be very useful for identifying potential biomarkers and studying molecular details of mammalian testis physiology.

## Authors' contributions

KKA conceptualized the project, assigned tasks to other team members, obtained funding, trained the team, contributed to validation of biocuration work, solved difficult cases of biocuration, identified the database components and designed broad structure, coordinated the events, supervised the progress of the work of the entire team and wrote the manuscript. DSC created the database and the associated software, and interfaced with biocurators. HS and VM also contributed towards database creation. NC performed biocuration, contributed significantly in training members and coordinating biocuration and data-validation efforts, and also contributed to the design of the database and its validation. SKS, DH, AB, SD, PB and LR contributed to biocuration, data collection and cross-validations. SKS, DH, AB and SD also contributed to manuscript writing. All authors read and approved the final manuscript.

## Supplementary Material

Additional file 1 **Notes S1**. Details of gene-set collection and fixed vocabulary usage in the database creation. Click here for file

Additional file 2 **Table S1**. Illustration of scoring derived from multiple data sets for specific ESLCs ('transcribed' or 'dormant' in normal human adult testis). Click here for file

Additional file 3 **Figure S1**. Schematic representation of creation and functioning of MGEx-Tdb. Click here for file

Additional file 4 **Table S2**. References and URLs for various databases/repositories used/referred in the study. Click here for file

Additional file 5 **Table S3**. Summary of manually curated data (MCD, from ***reports on individual gene studies***) for the genes selected for final comparison of databases. Click here for file

Additional file 6 **Table S4**. Scoring method for assessing the extent of agreement between the manually curated data (MCD, from ***reports on individual gene studies***) vs. the information from databases. Click here for file

Additional file 7 **Notes S2**. Procedure for comparison of MCD with the information from the databases. Click here for file

Additional file 8 **Table S5**. Procedure for relative assessment of amount and details of information, and agreement with reports from individual gene studies. Click here for file

Additional file 9 **Notes S3**. Procedure for comparison of results from various resources for gene expression under different physiological conditions and developmental stages in testis. Click here for file

Additional file 10 **Notes S4**. An overview of MGEx-Tdb use, with the help of screen-shots. Click here for file
