# Supplementary material for: A novel tissue-specific meta-analysis approach for gene expression predictions, initiated with a mammalian gene expression testis database
Source: BMC Genomics. 2010 Aug 11;11:467. doi: 10.1186/1471-2164-11-467 (PMC3091663; doi:10.1186/1471-2164-11-467)
Supplement: Additional file 1 — Notes S1. Details of gene-set collection and fixed vocabulary usage in the database creation. [file 1471-2164-11-467-S1.PDF]

Additional file 1

Notes S1: Details of gene-set collection and fixed vocabulary usage in the database creation:

An example to illustrate the segregation of composite data-sets into smaller and specific gene-sets.

Composite data-set indicating differentially expressed genes in hypogonadal vs. FSH-treated hypogonadal mice testes (Sadate-Ngatchou et al., 2004). This study originally reported eight gene-lists: two from untreated condition, two each from 4 and 8 hours post-treatment, and one gene-list each from 12 and 24 hours post-treatment. These gene-lists could be split into a more specific set of 15 gene-lists, as indicated below.

The gene-lists corresponding to specific duration of post-FSH treatment were processed and divided into the following categories:

- Definitely transcribed following FSH treatment (genes with identical transcription status in two lists were combined into one list)
- ‘may be transcribed’ (marginal level or contradictory transcription across two lists) following FSH treatment
- not transcribed following FSH treatment

Similarly, the untreated set of genes was used to derive three types of gene-lists.

- Definitely transcribed in untreated condition
- may be transcribed in untreated condition
- not transcribed in untreated condition

Identifying specific conditions, cell types etc as part of ‘fixed vocabulary’ usage in database creation.

A fixed vocabulary was developed to identify comparable conditions as described below.

Case 1: All data corresponding to sperm were collected under one name irrespective of equivalents (e.g., spermatozoa, sperms) used in articles/repositories. Similarly, since it is difficult to differentiate Leydig cells from other interstitial cells, the corresponding data were always entered under the fixed phrase ‘interstitial cells’, not specified as Leydig by the author.

Case 2: When collecting data from mice at different ages such as days 1, 4, 8, 11, 14, 18, 21, 26, 29, and 60 (adult) postpartum, we identified age groups into 3 broad categories: day 0-20 as post-natal mice, day 21-29 as young mice and day 30 onwards as an adult. The post-natal day mice were further classified into different categories based on the type of germ cells present at different ages: 0-6, 7-9, 10-17 and 18-20 days old *(J Cell Biol. 74:68-85)*. This would help in scoring as well as retrieving appropriate information for the users of the database. However, the original details were always retained.

Case 3: The ‘normal’ is the term used when the testis tissue or cell types was/were not treated in any way and is totally free of any testicular disorder. Any genetically modified organism was not considered as control, even if the author(s) prefer such reference. Such planned use of

vocabulary would enable a differential, but meaningful consensus expression status comparisons across conditions.
