# Supplementary material for: A novel tissue-specific meta-analysis approach for gene expression predictions, initiated with a mammalian gene expression testis database
Source: BMC Genomics. 2010 Aug 11;11:467. doi: 10.1186/1471-2164-11-467 (PMC3091663; doi:10.1186/1471-2164-11-467)
Supplement: Additional file 2 — Table S1. Illustration of scoring derived from multiple data sets for specific ESLCs ('transcribed' or 'dormant' in normal human adult testis). [file 1471-2164-11-467-S2.PDF]

Additional file 2

Table S1: Illustration of scoring derived from multiple data sets for specific ESLCs ('transcribed' or 'dormant' in normal human adult testis).

**Gene**	**Scores across multiple datasets (DS)**								**Final score (& expression status)**
	**DS1**	**DS2**	**DS3**	**DS4**	**DS5**	**DS6**	**DS7**	**DS8**	
HSPA1L	**2**(T)	**2**(T)	**2**(T)	**2**(T)	**2**(T)	*NA*	*NA*	*NA*	**10 (T)**
CCBP2	*NA*	*NA*	*NA*	*NA*	*NA*	**2**(D)	**2**(D)	**2**(D)	**6 (D)**
ZNF403	**2**(T)	**2**(T)	**2**(T)	**2**(T)	**2**(T)	**2**(D)	**2**(D)	*NA*	**6 (T)**

NA: Not Applicable (No data set for the respective ESLC),

T: Transcribed; **D**: Dormant
