# Supplementary figures and images for: A novel tissue-specific meta-analysis approach for gene expression predictions, initiated with a mammalian gene expression testis database
Source: BMC Genomics. 2010 Aug 11;11:467. doi: 10.1186/1471-2164-11-467 (PMC3091663; doi:10.1186/1471-2164-11-467)

### Additional file 3

Figure S1: Schematic representation of creation and functioning of MGEx-Tdb.

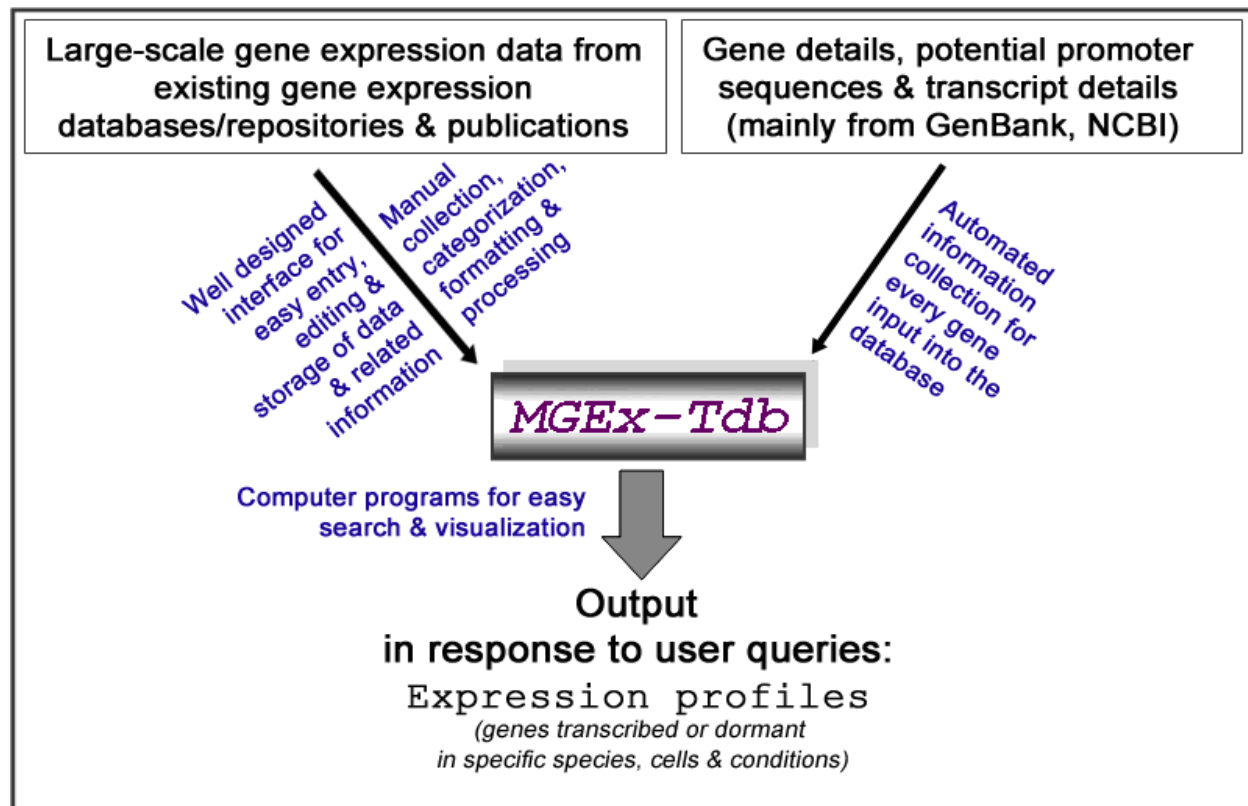

Supplement: Additional file 3 — Figure S1. Schematic representation of creation and functioning of MGEx-Tdb. [file 1471-2164-11-467-S3.PDF]
