# Supplementary material for: A novel tissue-specific meta-analysis approach for gene expression predictions, initiated with a mammalian gene expression testis database
Source: BMC Genomics. 2010 Aug 11;11:467. doi: 10.1186/1471-2164-11-467 (PMC3091663; doi:10.1186/1471-2164-11-467)
Supplement: Additional file 4 — Table S2. References and URLs for various databases/repositories used/referred in the study. [file 1471-2164-11-467-S4.PDF]

Additional file 4

Table S2: References and URLs for various databases/repositories used/referred in the study.

Databases	Uniform Resource Locator (URL)	References
UniGene	http://www.ncbi.nlm.nih.gov/unigene	Boguski and Schuler ('95)
BioGPS	http://biogps.gnf.org	Su et al. (2002)
RefExA	http://157.82.78.238/refexa/main_search.jsp	----
TissueDistributionDBs	http://genome.dkfz-heidelberg.de/menu/tissue_db	----
HPRD	http://www.hprd.org	Keshava et al. (2009)
GermSAGE	http://germsage.nichd.nih.gov	Lee et al. (2009)
GEO	http://www.ncbi.nlm.nih.gov/geo	Barrett et al. (2009)
ArrayExpress	http://www.ebi.ac.uk/microarray-as/ae	Parkinson et al. (2009)
Oncomine	http://www.oncomine.org	Rhodes et al. (2004)
TranscriptomeBrowser	http://tagc.univ-mrs.fr/tbrowser	Lopez et al. (2008)
Genevestigator	https://www.genevestigator.com	Hruz et al. (2008)
COXPRESdb	http://coxpresdb.jp	Obayashi et al. (2008)
SMD	http://smd.stanford.edu	Demeter et al. (2007)
CIBEX	http://cibex.nig.ac.jp/index.jsp	Ikeo et al. (2003)
Swiss-Prot	http://ca.expasy.org/sprot	Boeckmann et al. (2003)
dbTSS	http://dbtss.hgc.jp	Wakaguri et al. (2008)
Bgee	http://bgee.unil.ch/bgee	Bastian et al. (2008)
MGI (GXD)	http://www.informatics.jax.org/expression.shtml	Smith et al. (2007)
MRG	http://mrg.genetics.washington.edu	Charles et al. (2009)[1]
4DXpress	http://4dx.embl.de/4DXpress	Yannick et al. (2008)
TiGER	http://bioinfo.wilmer.jhu.edu/tiger/	Liu et al. (2008)
MatrixDB	http://matrixdb.ibcp.fr/	Chautard et al. (2009)
CleanEx	http://www.cleanex.isb-sib.ch/	Praz et al. (2009)
TiSGeD	http://bioinf.xmu.edu.cn/databases/TiSGeD/index.html	Xiao et al. (2010)

[1]Poster [Biol Reprod 81: 222. (2009)]

Notes:

1. Most of these sources as well as few other databases were considered for preliminary comparisons with MGEx-Tdb. Databases that give expression information only for a specific cell type (e.g., GermSAGE - for germ cells) or for a particular condition (e.g., Oncomine – for cancer), or those without feasible query features [e.g., Gene Expression Atlas of ArrayExpress and TranscriptomeBrowser] were ignored from the final comparison.
2. Databases that were omitted from the study include:
   - those (e.g., MatrixDB) that retrieve expression information from other gene expression databases, which have been already considered, and do not provide significant additional value; and
   - the ones (e.g., CleanEx) where the user has to compile a lot of information to determine the expression status for the queried gene, and still do not provide added advantage compared to other databases considered for final comparison here.
3. For a more detailed comparison, 23 commonly used and comparable gene expression databases have been taken up for various purposes. Results for some of these databases, which performed better than others, are displayed in the main manuscript (see results, tables 2-5, in the manuscript). Results for others have not been shown. For example, TiGER had only 27% agreement with the MCD and this was far below than the other widely used EST database, UniGene (46% agreement). Similarly, GXD, a database specific for mouse, had a lower coverage even for mouse than the MGEx-Tdb. Hence, it was not

considered for overall coverage comparisons, even though it was considered for comparison of mouse developmental stage information retrieval.

4. The recently published database (TiSGeD) gives the expression profile for many tissues. However, to determine the expression information of a gene, from this database, the user has to compile the data from different studies and probes. A study was performed to evaluate the performance of TiSGeD. It scored lesser than MGEx-Tdb in the amount and details of information availability (30%) and its agreement with literature (36%).
5. Databases on testis developed earlier but not accessible/functional during our study-period:
   (a) TestisBank: Claussen et al., 2002. TestisBank: an internet-based gene sequence database of the testis. Int J Androl 25:175-179. (URL: http://medweb.uni-muenster.de/TestisBank/)
   (b) dbTEST: A Database of Transcripts Expressed in Spermatogenesis and Testis. (Halgren et al. unpublished; URL: http://www.bch.msu.edu/~zacharet/dbtest/)
