# Supplementary material for: A novel tissue-specific meta-analysis approach for gene expression predictions, initiated with a mammalian gene expression testis database
Source: BMC Genomics. 2010 Aug 11;11:467. doi: 10.1186/1471-2164-11-467 (PMC3091663; doi:10.1186/1471-2164-11-467)
Supplement: Additional file 5 — Table S3. Summary of manually curated data (MCD, from reports on individual gene studies) for the genes selected for final comparison of databases. [file 1471-2164-11-467-S5.PDF]

Additional file 5

Table S3: Summary of manually curated data (MCD, from reports on individual gene studies) for the genes selected for final comparison of databases.

Genes	Expressed in	Number of articles used to extract expression information
RBM	Testis only	5
MAGEB1	Testis only	5
TSPY1	Testis only	4
ACRV1	Testis only	4
PRM1	Testis only	2
SSX1	Testis only	3
SPACA3	Testis & pancreas	2
DDX4	Testis & ovary	2
RAD9B	Testis & Skeletal muscle	2
BEX1	Ubiquitous	2
PIWIL1	Mainly testis (low in other tissues)	4
ARF6	Ubiquitous	2
BMI1	Ubiquitous	2

Note:
Statistics of the type of techniques encountered in literature during biocuration: 12 Northern blot, 12 RT-PCR, 1 quantitative end-point RT-PCR, 1 semi-quantitative PCR, 1 quantitative RT-PCR, 2 ISH, 2 multi tissue expression array, 1 multiple tissue cDNA panel, 1 RNA master blot, 1 Microarray, 9 IHC, 3 Western blot, 1 *in situ* immunostaining, 1 Immunofluorescence, 1 Immunolocalization and 1 IHC using tissue microarray.
