# Supplementary material for: A novel tissue-specific meta-analysis approach for gene expression predictions, initiated with a mammalian gene expression testis database
Source: BMC Genomics. 2010 Aug 11;11:467. doi: 10.1186/1471-2164-11-467 (PMC3091663; doi:10.1186/1471-2164-11-467)
Supplement: Additional file 6 — Table S4. Scoring method for assessing the extent of agreement between the manually curated data (MCD, from reports on individual gene studies) vs. the information from databases. [file 1471-2164-11-467-S6.PDF]

Additional file 6

Table S4: Scoring method for assessing the extent of agreement between the manually curated data (MCD, from reports on individual gene studies) vs. the information from databases.

MCD for different tissues	Gene expression status from databases and its agreement with MCD	Score
For testis tissue only	Agreement with MCD	1
	Disagreement with MCD	-1
	No information on testis expression	0
For other tissues	Agreement with MCD expression statement	1
	Disagreement with MCD expression statement	
	≤10% disagreement	0
	11-20% disagreement	-0.25
	21-30% disagreement	-0.5
	31-40% disagreement	-0.75
	>40% disagreement	-1
	No information on other tissues expression	0
No gene or expression details		0

Note:

For example, MCD gave the expression of a gene 'X' in 10 different tissues and if the expression profile retrieved from the database for that gene, contradicted with MCD in 1 of the 10 tissues, then no contradiction score is given to database. If the contradiction was in 2-3 tissues, then a penalty of '-0.5', and for >4 tissues, a penalty of '-1' was given.
