# Supplementary material for: A novel tissue-specific meta-analysis approach for gene expression predictions, initiated with a mammalian gene expression testis database
Source: BMC Genomics. 2010 Aug 11;11:467. doi: 10.1186/1471-2164-11-467 (PMC3091663; doi:10.1186/1471-2164-11-467)
Supplement: Additional file 7 — Notes S2. Procedure for comparison of MCD with the information from the databases. [file 1471-2164-11-467-S7.PDF]

Additional file 7

Notes S2: Procedure for comparison of MCD with the information from the databases:

The comparison of MCD with the information from the databases was performed independently by two individuals. There was a difference of 1.67 %, on an average, between the final scores derived by the two researchers. Average number of abstracts screened for each gene was 185 and number of full text read for each gene was 12. The average number of relevant papers with useful information was 3, while that for experiments with supporting evidence for the expression status, was 4. A total of 34 experimental evidences were at the mRNA level while 16 were at the protein level.
