# Supplementary material for: A novel tissue-specific meta-analysis approach for gene expression predictions, initiated with a mammalian gene expression testis database
Source: BMC Genomics. 2010 Aug 11;11:467. doi: 10.1186/1471-2164-11-467 (PMC3091663; doi:10.1186/1471-2164-11-467)
Supplement: Additional file 8 — Table S5. Procedure for relative assessment of amount and details of information, and agreement with reports from individual gene studies. [file 1471-2164-11-467-S8.PDF]

Additional file 8

Table S5: Procedure for relative assessment of amount and details of information, and agreement with reports from individual gene studies.

Information availability			Volume of supporting data	
Tissue/cell type	***Expression information***	***Score***	***No. of resources used to determine the expression of a gene***	***Score***
Testis	Expressed/Not Expressed	0.5	1	0.1
	No information	0	2	0.2
Cell types	Expressed/Not Expressed	0.5	3	0.3
	No information	0	>3	0.5
Conditions	Expressed/Not Expressed	0.5		
	No information	0		

Agreement with literature (reports on individual gene studies)

Tissue/cell type	***Expression information***	***Score***
Testis	Expressed	0.5
	Not expressed	-0.5
	No information	0
Cell types	Expressed	0.5
	Not expressed	-0.5
	No information	0

Note:

1. The scoring was done separately for human, mouse and rat species. The average score for each of the species is given in table 3.

   For example***:*** If prm2 was expressed in spermatid and spermatozoa according to HPRD, a score of 0.5 was given for the expression information. This information was obtained from 2 papers and hence a score of 0.2 was given for the volume of supporting data. According to our manual curation, we found that prm2 is a germcell specific marker. Hence, an additional score of 0.5 was given to the database for agreeing with our collected information.
2. List of genes that are predominantly expressed in mammalian testicular cells considered for the analysis: DAZL, Vim, Sycp1, PUM2, Tex101, DPPA3, PRM2, MAGEA4, PGK2, INSL3
