# Supplementary material for: A novel tissue-specific meta-analysis approach for gene expression predictions, initiated with a mammalian gene expression testis database
Source: BMC Genomics. 2010 Aug 11;11:467. doi: 10.1186/1471-2164-11-467 (PMC3091663; doi:10.1186/1471-2164-11-467)
Supplement: Additional file 9 — Notes S3. Procedure for comparison of results from various resources for gene expression under different physiological conditions and developmental stages in testis. [file 1471-2164-11-467-S9.PDF]

Additional file 9

Notes S3: Procedure for comparison of results from various resources for gene expression under different physiological conditions and developmental stages in testis:

The databases that allow querying for gene expression using any bio-entity, other than gene/protein name, were compared using different examples. These case studies were: *azoospermia, asthenozoospermia & "testicular cancer" in humans, chemical/ drug treatment (adjudin) in rat & developmental stages (postnatal) in mouse*. Testis, a*zoospermia* & *asthenozoospermia* terms were used as keyword(s) to query theses databases. In case of *"testicular cancer"*, databases were queried with the exact phrase (BioGPS, UniGene), or using boolean operators (*testis AND cancer*, in case of UniGene & RefExA) or using keywords/disease names (TissueDistributionDBs & HPRD), as suggested by the respective database. Databases were also queried with *adjudin* for the chemical/drug treatment and *postnatal* for the developmental stages. Databases restricted to *Homo sapiens* (HPRD & RefExA) were not considered in the above two cases.

For comparison with databases that specifically provide expression information for developmental stages in mouse, "postnatal", "0-6 days" (specific among post-natal phases) or alternative equivalent stages e.g., Theiler stage (TS) were considered.

Note: Mouse embryos can be staged according to a method described by Theiler (1989, The House Mouse: Atlas of Embryonic Development. New York: Springer-Verlag).
