# Supplementary material for: A novel tissue-specific meta-analysis approach for gene expression predictions, initiated with a mammalian gene expression testis database
Source: BMC Genomics. 2010 Aug 11;11:467. doi: 10.1186/1471-2164-11-467 (PMC3091663; doi:10.1186/1471-2164-11-467)
Supplement: Additional file 10 — Notes S4. An overview of MGEx-Tdb use, with the help of screen-shots. [file 1471-2164-11-467-S10.PDF]

Additional file 10

Notes S4: An overview of MGEx-Tdb use, with the help of screen-shots.

Home page of MGEx-Tdb:

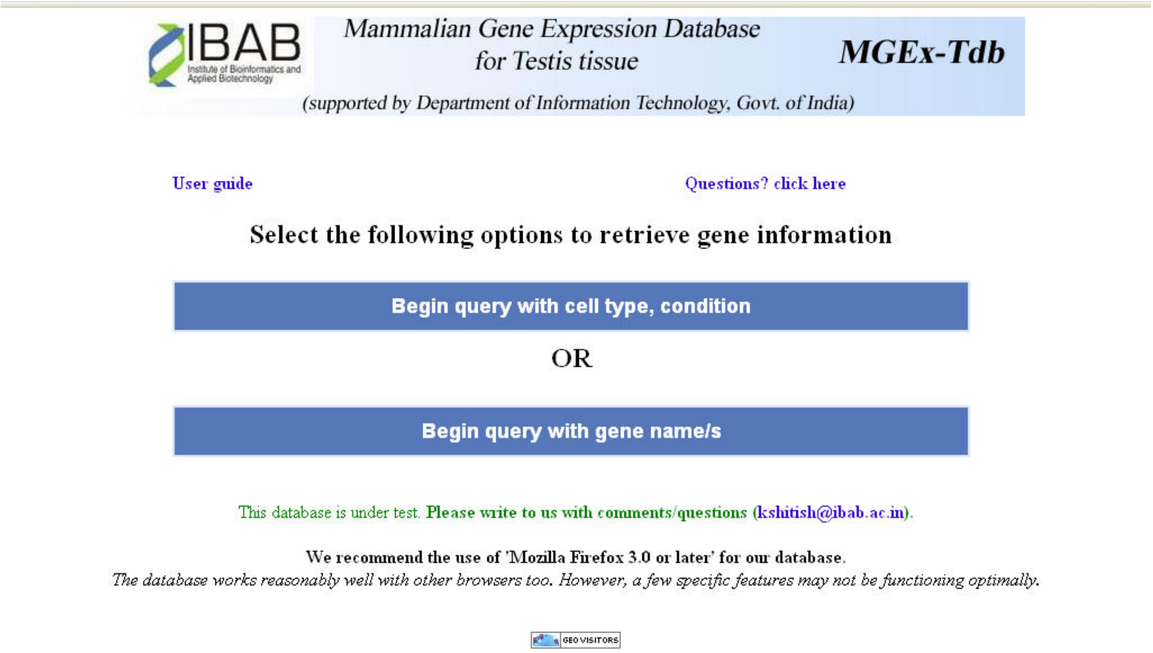

Query with cell type and condition:

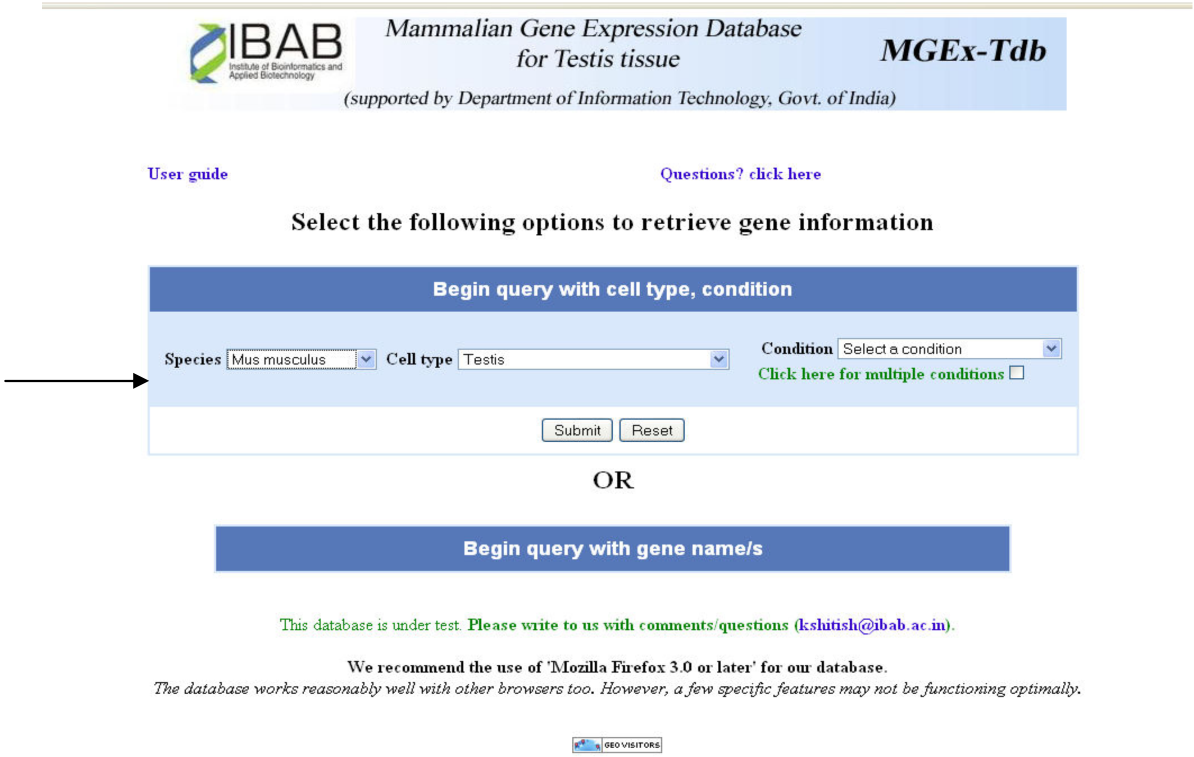

Query database to get list of genes expressed/dormant in normal human testis:

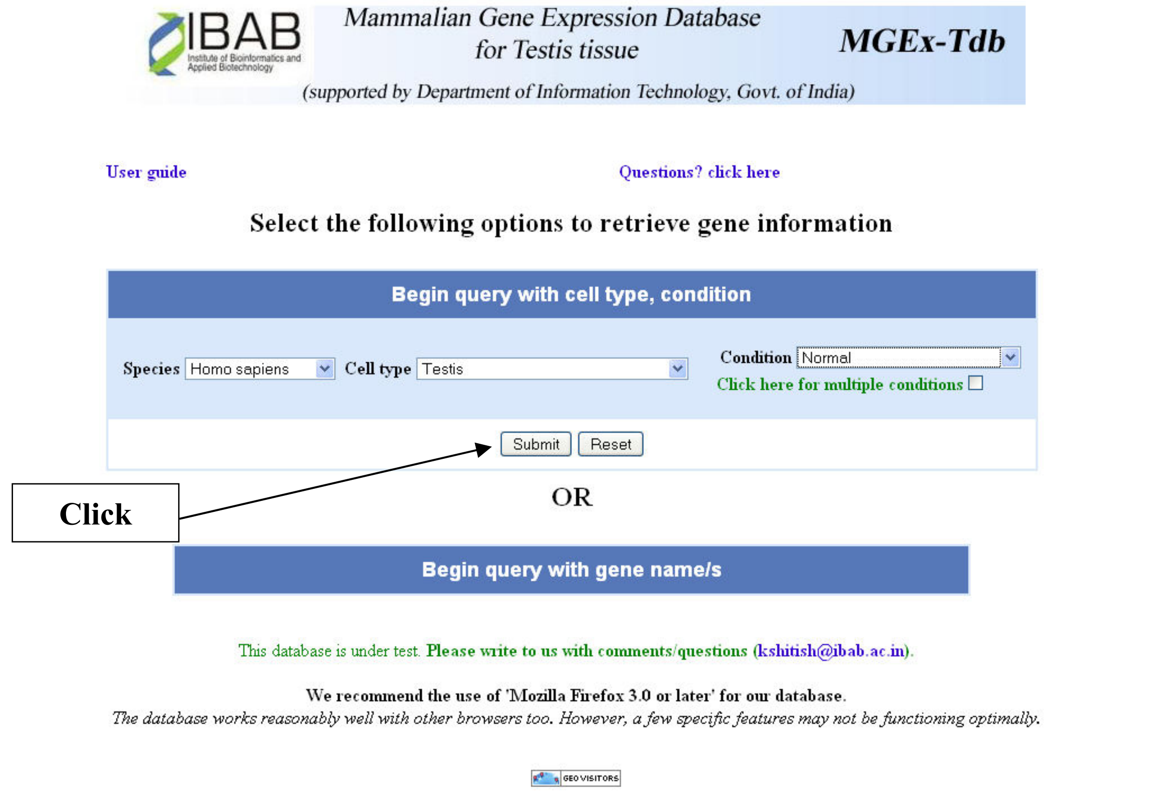

List of genes transcribed/dormant with reliability score in normal human testis:

IBAB Institute of Bioinformatics and Applied Biotechnology

Mammalian Gene Expression Database
for Testis tissue

MGEx-Tdb

(supported by Department of Information Technology, Govt. of India)

Home   Questions? click here

Genes in Homo sapiens in Testis
Normal condition

Top 20 Hits are shown below

Click →

Transcribed			Dormant		
Serial no.	Gene name	Reliability score	Serial no.	Gene name	Reliability score
1	GAGE2A	8.00	1	HIST1H1T	2.00
2	ACRV1	6.00	2	PLAUR	2.00
3	PRM1	6.00	3	KLK13	2.00
4	SPA17	6.00	4	SHBG	2.00
5	GK2	6.00	5	POMC	2.00
6	ADAM2	6.00	6	BRSK2	2.00
7	BRDT	6.00	7	SEC31B	2.00
8	JARID1B	6.00	8	RAD51	2.00
9	FATE1	6.00	9	MYCL2	2.00
10	CETN1	6.00	10	ARID4B	2.00
11	SOX30	6.00	11	RAI14	2.00
12	IL13RA2	6.00	12	STON1	2.00
13	MLF1	6.00	13	CDH13	2.00
14	BUB1	6.00	14	KIF22	2.00
15	RANBP5	6.00	15	CDC25A	2.00

Information about GAGE2A:

IBAB Institute of Bioinformatics and Applied Biotechnology

Mammalian Gene Expression Database
for Testis tissue

MGEx-Tdb

(supported by Department of Information Technology, Govt. of India)

Home   Questions? click here

G antigen 2A *(Homo sapiens)* -GAGE2A	Gene Information	Transcript Information	Protein Information	Promoter Information
	Expression Information: tissue and/or cell, condition		Relevant PubMed Papers	Other Information

Gene information about GAGE2A:

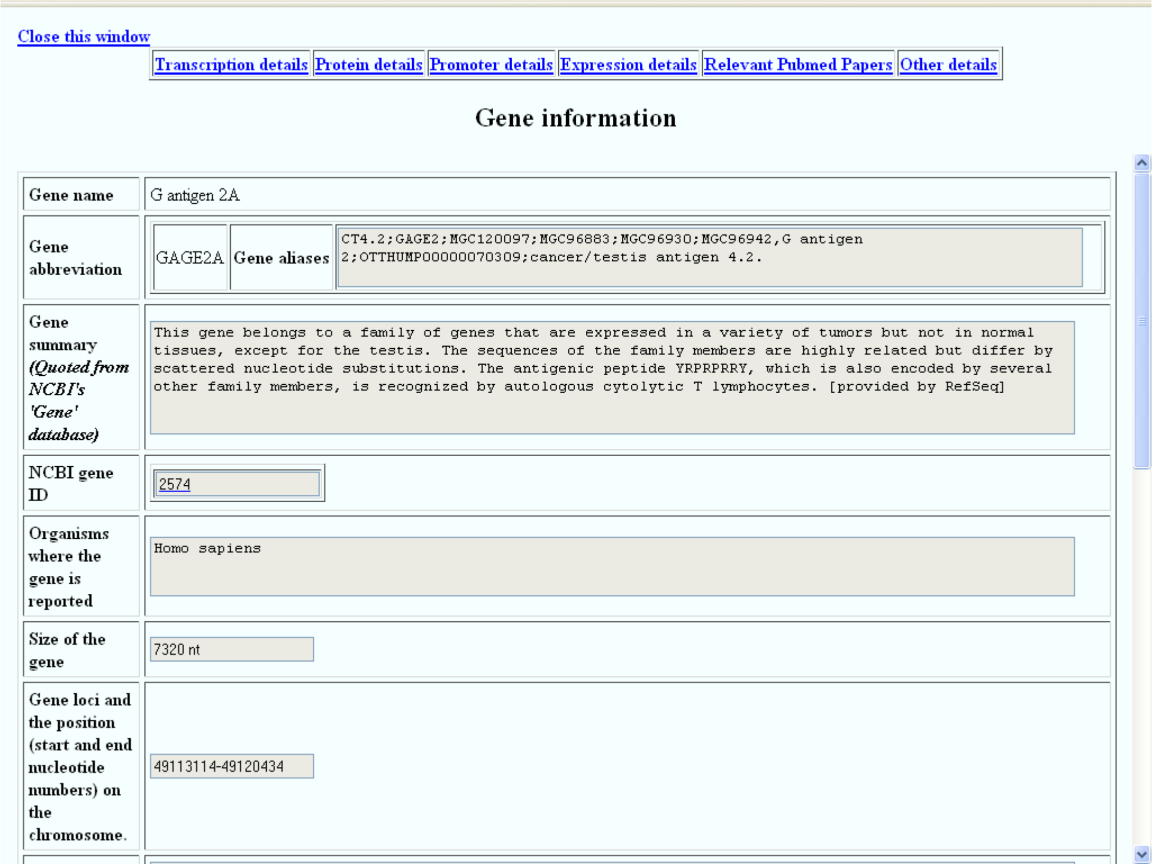

Transcript information for GAGE2A:

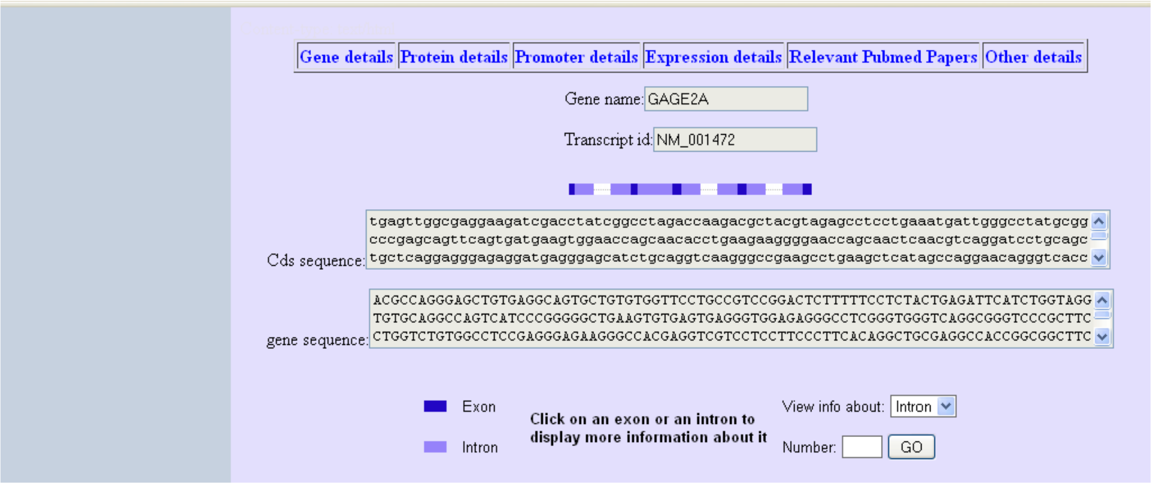

Expression information of GAGE2A:

Close this window

Transcription details | Protein details | Promoter details | Gene details | Relevant PubMed papers | Other details

Expression information

Manually curated and automatically processed data from genome-wide studies for GAGE2A *(Homo sapiens)*

Species	Cell type	Sub Celltype	Condition	SubCondition	Expression Status	Reliability Score
Homo sapiens			Normal		Transcribed	8.00
Homo sapiens			Normal (Adult)		Marginal	0.00
Homo sapiens			Disease	Infertility - Azoospermia	Marginal	0.00
Homo sapiens			Disease	Infertility - Obstructive Azoospermia	Marginal	0.00
Homo sapiens			Disease	Infertility - Non Obstructive Azoospermia	Marginal	0.00
Homo sapiens			Disease	Cancer	Marginal	0.00
Homo sapiens			Developmental Stages	Embryonic stage	Marginal	0.00
Homo sapiens	Somatic Cell	Leydig	Normal (Adult)		Marginal	0.00
Homo sapiens	Germ Cell	Sperm	Normal (Adult)		Marginal	0.00

Query with gene name (eg:ACRV1):

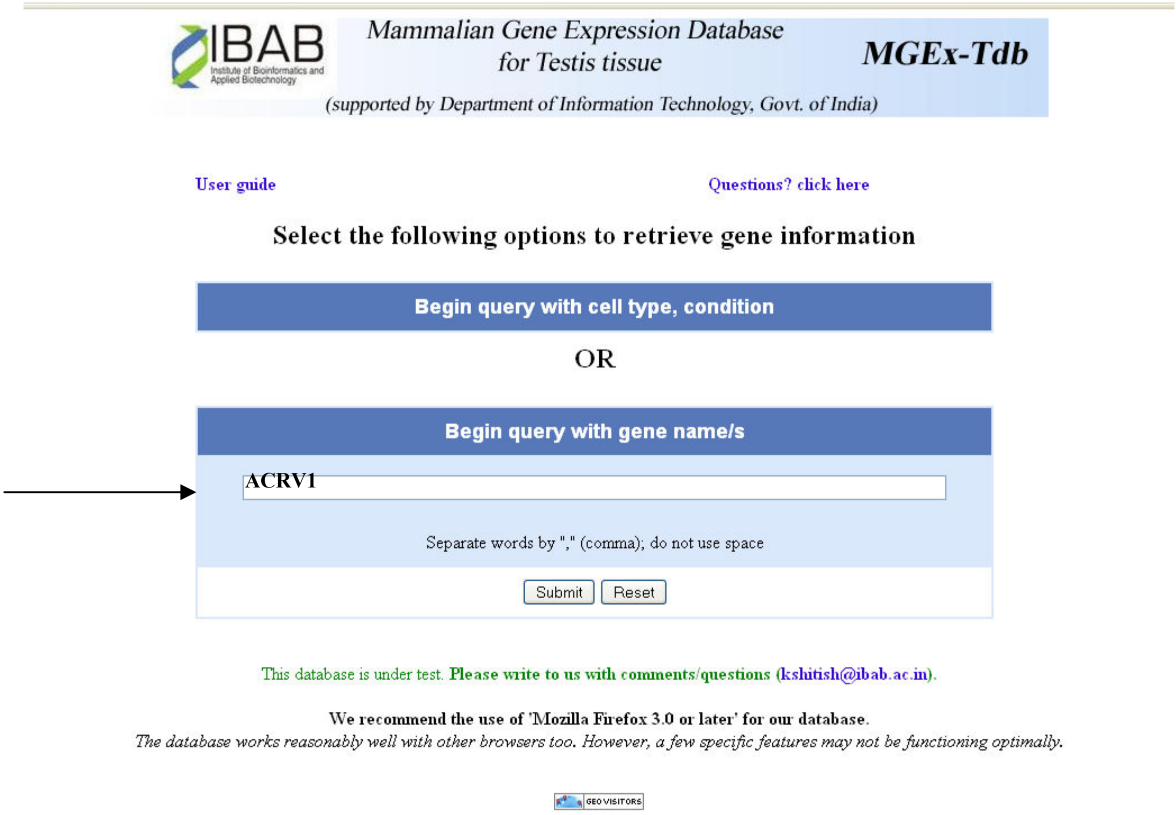

Result page for ACRV1:

Home

Questions? click here

ACRV1

- Identical matches
- Partial matches

Identical Matches for ACRV1: 3 hits found

TOP

acrosomal vesicle protein 1 *(Homo sapiens)* - ACRV1	Gene Information	Transcript Information	Protein Information	Promoter Information
	Expression Information: tissue and/or cell, condition		Relevant PubMed Papers	Other Information
acrosomal vesicle protein 1 *(Mus musculus)* - Acrv1	Gene Information	Transcript Information	Protein Information	Promoter Information
	Expression Information: tissue and/or cell, condition		Relevant PubMed Papers	Other Information
acrosomal vesicle protein 1 *(Rattus norvegicus)* - Acrv1	Gene Information	Transcript Information	Protein Information	Promoter Information
	Expression Information: tissue and/or cell, condition		Relevant PubMed Papers	Other Information

Partial Matches for ACRV1: 0 hits found

TOP
